# Stronger Empathy and Better Peer Relationship? One‐Year Cross‐Lagged Panel Analysis in Preschoolers

**DOI:** 10.1002/pchj.815

**Published:** 2024-11-28

**Authors:** Xiao Zeng, Yao Xiong, Kainian Mo, Mengyao Yang, Binglin Xie, Zhiqiang Yan

**Affiliations:** ^1^ School of Education Hunan First Normal University Changsha China; ^2^ Department of Psychology Hunan Normal University Changsha China; ^3^ Cognition and Human Behavior Key Laboratory of Hunan Province Hunan Normal University Changsha China

**Keywords:** cross‐lagged analysis, empathy, peer relationship, preschooler, social network analysis

## Abstract

The development of empathy and the establishment of peer relationships significantly influence the quality of preschoolers' social lives. Past research consistently demonstrates a strong correlation between empathy and peer relationships. However, debates persist regarding causality. To provide evidence on this matter, we conducted a year‐long longitudinal study involving 160 preschoolers at T1, with 137 children (mean age = 60.99 months, *SD* = 3.51; 66 males) retained at T2, 1 year later. Our study employed a two‐wave design and cross‐lagged panel analysis. Data on empathy and peer relationships were collected through empathy story tasks and peer nominations. For the analysis of peer nominations, we selected three centrality measures from social network analysis: degree centrality (number of direct friendships), closeness centrality (proximity between network members), and betweenness centrality (control/mediation of information exchange). Results from the cross‐lagged panel analysis reveal that preschoolers' affective and cognitive empathy at T1 positively and significantly predict peer relationships at T2. Specifically, both forms of empathy predict degree centrality and closeness centrality. Additionally, hierarchical linear modeling indicates that, even after controlling for affective empathy, cognitive empathy significantly predicts both degree centrality and closeness centrality at T2. These findings underscore the importance of nurturing empathy, especially cognitive empathy, to enhance peer relationships among preschoolers. Consequently, educators can play a vital role in improving peer relationships by intervening in empathy development, promoting greater social interaction.

## 1 Introduction

Interpersonal interactions play a pivotal role, distinct from the familial environment, in shaping the social and emotional development of preschoolers (Torres, Domitrovich, and Bierman 2015). Therefore, identifying the key factors influencing social interaction, particularly peer relationships among preschoolers, is crucial. Empirical evidence suggests that empathy has the potential to foster peer relationships (Van Ryzin and Roseth 2019). Empathy includes both affective and cognitive components (Zaki 2016), which facilitate the perception and understanding of emotional cues from others. A recent three‐level meta‐analysis focused on preschoolers has revealed a moderate correlation between empathy and peer relationships (Yan, Zhou, and Liu 2022). However, the exact nature of this relationship remains unclear, particularly regarding whether affective and cognitive empathy play distinct roles. Moreover, previous studies (Van Ryzin and Roseth 2019, 2022) have shown significant bidirectional predictions between peer relationships and affective empathy, but these studies have primarily focused on peer relationships themselves. To address this gap, we conducted a 1‐year longitudinal study with preschoolers, aiming to explore the associations between both affective and cognitive empathy and peer relationships. Our use of social network analysis represents a notable innovation, transitioning from static data collection to the dynamic mapping of social networks.

### 1.1 Empathy and Its Development

Although definitions of empathy may vary (de Waal 2008; Yan, Zhou, and Liu 2022), it is widely recognized as the ability to perceive and understand the emotions of others (Decety et al. 2015). Empathy is typically divided into two components: affective empathy and cognitive empathy (Yan et al. 2020). Affective empathy is an innate ability (Huang and Su 2012) that functions as an automatic emotional response mechanism (de Waal and Preston 2017; Gamble et al. 2024), allowing individuals to perceive and share the emotions of others (Eisenberg and Strayer 1987). Following the development of affective empathy, cognitive empathy emerges as a higher order function involving the capacity to adopt others' perspectives and understand their thoughts and intentions (de Waal 2008; Yan et al. 2020). Bensalah, Caillies, and Anduze (2015) distinguish between two forms of cognitive empathy that arise as early as the preschool years: other‐focused role‐taking and self‐focused role‐taking (Hoffman 2000).

A notable observation is that affective and cognitive empathy follow distinct developmental trajectories before the age of six. A review of previous research indicates that while affective empathy is intrinsic and tends to a plateau or even diminish with age (Decety and Svetlova 2012), cognitive empathy follows an upward trajectory as children grow older (Bensalah, Caillies, and Anduze 2015). Affective empathy, being innate, enables infants to automatically respond to external emotional cues. For example, newborns often display increased crying when exposed to the cries of other infants, an alternate emotional experience (Dondi, Simion, and Caltran 1999; Martin and Clark 1982). In infancy, infants also engage in automatic imitation of others' emotional expressions (Harrison, Morgan, and Critchley 2010; Kaitz et al. 1988). Additionally, preschoolers unconsciously mimic others' emotional expressions (Skinner et al. 2020). When they observe a stranger displaying warm, friendly nonverbal signals toward another person, they are more likely to form positive attitudes toward that individual, compared with those who receive cold, unfriendly signals (Skinner et al. 2020).

In contrast, cognitive empathy develops gradually after birth and plays a critical role in helping children recognize and understand the emotions and perspectives of others (Yan et al. 2020). Some researchers, using nonverbal research paradigms such as gaze preference and violation‐of‐expectation tasks, have found that infants as young as 6 months old show a preference for individuals exhibiting helpful behavior, which is considered an early manifestation of cognitive empathy (Hamlin, Wynn, and Bloom 2007). Studies also indicate that the cognitive developments occurring during the preschool years are linked to both cognitive and behavioral forms of empathy (Bensalah, Caillies, and Anduze 2015). Nonetheless, meta‐analytic evidence suggests that affective empathy remains the dominant component in preschoolers (Yan and Su 2021).

### 1.2 Peer Relationship and Social Network Analysis

Peer relationships refer to the interpersonal connections established and cultivated through communication among individuals at similar levels of psychological development (Hay, Payne, and Chadwick 2004; Holloway and Reichhart‐Erickson 1989). For preschoolers, interactions with peers are primarily centered around building relationships within the classroom environment, making classmates a crucial factor in the development of peer relationships (Fujisawa, Kutsukake, and Hasegawa 2009).

A common method for assessing peer relationships is peer nomination (Coie and Dodge 1983), which can be analyzed using various techniques. One prominent approach is social network analysis, which not only characterizes an individual's peer relationships but also provides a detailed understanding of their position within a broader social structure (Borgatti et al. 2009). Social network analysis includes three key metrics for evaluating individual peer relationships: degree centrality, betweenness centrality, and closeness centrality (Butts 2008; Lee et al. 2022). Degree centrality measures the number of connections an individual has and is the most widely used metric. Closeness centrality assesses the average distance between an individual (node) and all other individuals in the network, offering insights into how well‐connected they are overall. Betweenness centrality, on the other hand, evaluates an individual's ability to connect disparate individuals, groups, or subgroups within the network, with those high in betweenness centrality serving as key connectors.

In summary, social network analysis is a valuable tool for mapping the peer relationships of preschoolers in a classroom setting. This study, therefore, employs social network analysis to examine preschoolers' peer relationships.

### 1.3 Peer Relationship to Empathy or Empathy to Peer Relationship

While a positive correlation between peer relationships and empathy has been established, further clarification is required to understand the directionality of this relationship. Meta‐analytic evidence has demonstrated a significant positive association between peer relationships and empathy (Boele et al. 2019). Notably, the strength of this association varies across the lifespan, with its peak occurring in mid‐childhood, followed by preschool and adolescence, and gradually weakening in early adulthood, forming an inverted U‐shaped trajectory (Yan, Zhou, and Liu 2022). Given the distinct developmental trajectories of peer relationships and empathy, it is essential to explore how these two factors relate, particularly within the context of preschool children.

Most researchers maintain that empathy plays a crucial role in fostering peer relationships. From a developmental perspective, social cognition emerges earlier than interpersonal interactions. Studies suggest that empathy is innate (de Waal 2008), while peer relationships typically develop after children begin kindergarten. In terms of interpersonal interaction, high empathy—rather than low empathy—facilitates the formation of peer relationships. Preschoolers with heightened empathy are more socially adept, popular, and skilled at building positive peer relationships. Empathy enhances children's social competencies, further solidifying its independent role in shaping peer relationships (Fink and de Rosnay 2023). Specifically, affective empathy enables preschoolers to tune into and resonate with the emotions of others, while cognitive empathy allows them to understand the emotional states and perspectives of their peers. Both forms of empathy have been shown to enhance peer relationships (Caravita and Blasio 2009; Van Ryzin and Roseth 2022).

Therefore, we proposed H1 and H2:

*Affective empathy promotes peer relationships*.

*Cognitive empathy promotes peer relationships*.

Substantial evidence also highlights the significant role that peer relationships play in fostering the development of empathy. The Social Ecosystems Theory, proposed by Zastrow and Kirst‐Ashman (2003), conceptualizes the social environments in which individuals grow—comprising family units, communities, and other systems—as interrelated social ecological systems. This theory emphasizes the influence of peers within these environments on the social development of preschoolers (Gülay Ogelman, Seçer, and Önder 2015). Peer relationships, as an environmental factor, significantly influence the development of empathy. For instance, Van Ryzin and Roseth (2019) found that group collaboration and cooperative activities enhance interpersonal relationships, leading to improved understanding of both cognitive and emotional states in others, ultimately fostering greater empathy for peers' emotional well‐being.

We proposed H3 and H4:

*Peer relationships promotes affective empathy*.

*Peer relationships promotes cognitive empathy*.

In summary, the relationship between empathy and peer relationships remains an area of ongoing inquiry. On the one hand, empathy emerges early in life and contributes substantially to the formation of interpersonal connections, suggesting that it may drive the establishment of peer relationships. On the other hand, experiences within these relationships may, in turn, nurture the development of empathy. This suggests a reciprocal relationship, where empathy and peer relationships mutually influence one another. Accordingly, the present study employs a longitudinal design to explore the bidirectional relationship between empathy and peer relationships in preschool children, offering a unique perspective on their causal interplay.

## 2 Method

### 2.1 Participants

To evaluate our hypothesis, we conducted a 1‐year follow‐up study, encompassing the enrollment of 160 children at T1 and the retention of 137 children (mean age = 60.99 months, standard deviation of age = 3.51; 66 males) at T2. Participants were recruited from urban preschools in Changsha, Hunan Province, China. The follow‐up interval averaged 368 days for all retained participants. The demographic analysis indicates that there were no significant differences in age or gender between the children who completed the follow‐up assessment and those who did not (*p*s > 0.05). This study received ethical approval from the Ethics Committee of Hunan Normal University, and all parents provide consent for their children's participation. In adherence to the principles outlined in the Declaration of Helsinki, we initially furnished them with a written elucidation of the study and procured their signatures, signifying their authorization for their children's participation.

### 2.2 Measures

#### 2.2.1 Peer Relationship

Peer relationships were assessed through peer nominations and Social Network Analysis (SNA) (Coie and Dodge 1983; Huang and Su 2014; Butts 2008; Burt, Kilduff, and Tasselli 2013). In SNA, the fundamental units are nodes and edges, representing the structural and dynamic relationships within the network. Nodes signify individual participants, while edges depict the connections between them. In this study, each child is considered an independent node, connected through friendship ties (edges). Once the collection and coding of all nominations were completed, a comprehensive network map emerged.

Specifically, each child was individually asked to nominate up to three peers in response to the question, “Which children do you prefer to play with the most?” in a quiet kindergarten room. All peer nominations were obtained through free recall. Nominations were coded numerically; for example, if child A nominated B, C, and D, we coded this as 1–2, 1–3, and 1–4. Utilizing these nomination results, we conducted a social network analysis using the Pajek software (Burt, Kilduff, and Tasselli 2013; Mrvar and Batagelj 2016), which led to the computation of network centrality indices, including degree centrality, closeness centrality, and betweenness centrality.

Degree centrality is a metric that assesses an individual's importance based on the number of direct friendships they possess. It represents the total number of connections (edges) a node has and is calculated as the sum of all edges linked to that node. Closeness centrality measures significance by evaluating a node's ability to connect with others effortlessly. It indicates how close a node is to all other nodes in the network, calculated as the reciprocal of the sum of the shortest path distances from the node to all other nodes. Betweenness centrality quantifies an individual's importance by counting the number of shortest paths that pass through them between two other individuals. This measure reflects the extent to which a node lies on the shortest paths connecting other nodes and is determined by counting these paths that traverse the node.

#### 2.2.2 Empathy

To enhance ecological validity, this study employed a videotaped vignette empathy story task, as outlined by Yan, Pei and Su (2017). Each video portrayed a child interacting with one parent while expressing a range of intense emotions, including happiness, anger, sadness, and fear. Here is a summary of the four videos. Happiness: A young girl sings and dances in a room, receiving applause from her father after finishing her song. Anger: A child plays with a performance prop but is stopped by her father, who warns her about potential damage. Ignoring his advice, she has the prop confiscated and reacts by discarding her clothes. Sadness: A little girl, alone without her father nearby, cries and attempts to go outside to find him. She is hindered by her uncle due to safety concerns, and her distress intensifies. Fear: A group of boys and girls ventures into a dark cave, repeatedly asking if anyone is there. One girl falsely claims someone is behind them, leading to a collective panic and escape.

During this task, participants were instructed to watch the videos and then respond to a series of questions presented after each video. These inquiries included: “What's your feeling now?,” “Why do you feeling this way?,” “What do you think the child in the story feels like?,” and “Why do you think the child feels that way?” The first two questions are designed to assess affective empathy, while the latter two focus on cognitive empathy. Responses were evaluated using the empathy continuum scoring system developed by Strayer (1993), which assesses two dimensions: affective empathy and cognitive empathy. The score range for affective empathy is 0–3, reflecting the simplicity and immediacy of emotional responses. In contrast, cognitive empathy involves more complex reasoning and understanding, requiring a wider score range of 0–7 to capture the nuances of this process. The responses to each question were summed to obtain total scores for both affective and cognitive empathy. Two coders independently coded all the data, achieving an inter‐rater agreement of 0.87. Any discrepancies were resolved through discussion.

### 2.3 Procedure

Children were invited to participate in the study in a familiar setting and were asked to complete assignments in a balanced sequence. The first step involved measuring empathy. Following the protocol established by Strayer (1993), participants initially viewed pictures expressing happiness, anger, sadness, and fear. They were first asked to identify these four basic emotions from the pictures; correct identification indicated their ability to accurately describe and match emotions. Subsequently, participants were required to match these emotions after watching a video depicting various emotional scenarios and answer related questions. After the empathy assessment, the study proceeded to measure peer relationships. Drawing from previous research methodologies (Coie and Dodge 1983; Huang and Su 2014), participants responded to questions about their favorite friends, providing the names of up to three friends.

The follow‐up assessment, conducted after an average of 368 days, involved reevaluating participants through the videotaped vignette empathy story task and peer nominations.

### 2.4 Statistical Analysis

The analysis of the data was executed employing Mplus (version 7.4). To investigate the causal relationship between empathy and peer relationships, we conducted a two‐wave cross‐lagged panel analysis following the approach outlined by Kessler and Greenberg (1981). We denoted empathy and peer relationships at T1 as variables *x*1 and *y*1, respectively, and these at T2 were represented as *x*2 and *y*2. The establishment of a 95% confidence interval was accomplished through the utilization of a bootstrap method involving 1000 samples.

## 3 Results

First, the development of empathy and peer relationships was assessed through the utilization of paired samples *t* test (refer to Table 1). Affective empathy (*t* (136) = −4.13, *p* < 0.01, Cohen's *d* = −0.35) and cognitive empathy (*t* (138) = −7.10, *p* < 0.01, Cohen's *d* = −0.61) exhibited greater values at time 2 compared with time 1, indicating a notable increase over the observed period.

**TABLE 1:** Descriptive statistics and paired‐samples *t* test of empathy and peer relationship.

Variable	T1				T2				*t*
	*M*	SD	Min	Max	*M*	SD	Min	Max	
Affective empathy	6.93	2.21	0	11	7.92	2.79	0	12	−4.13***
Cognitive empathy	10.10	2.39	3	15	11.83	2.64	2	16	−7.10***
Degree centrality	5.99	2.73	3	18	6.04	2.26	3	14	−0.24
Closeness centrality	0.42	0.04	0.33	0.58	0.42	0.05	0.32	0.55	0.41
Betweenness centrality	0.05	0.04	0	0.17	0.06	0.05	0	0.26	−2.41*

*** *p* < 0.001.

* *p* < 0.05.

Subsequently, a correlation analysis was undertaken to examine the relationship between empathy and peer interactions (refer to Table 2). The findings elucidated that both affective empathy and cognitive empathy displayed positive correlations with degree centrality and closeness centrality from time 1 to time 2. Nonetheless, this trend did not manifest in the context of betweenness centrality.

**TABLE 2:** Correlation analysis of empathy and peer relationship across time.

Variable	1	2	3	4	5	6	7	8	9	10	11
1. T1 AFF											
2. T2 AFF	0.38***										
3. T1 COG	0.67***	0.42***									
4. T2 COG	0.28**	0.59***	0.36***								
5. T1 DC	0.27**	0.19*	0.29***	0.17*							
6. T2 DC	0.32***	0.34***	0.36***	0.34***	0.52***						
7. T1 CC	0.32***	0.24**	0.35***	0.23**	0.78***	0.42***					
8. T2 CC	0.32***	0.25**	0.30***	0.25**	0.31***	0.63***	0.41***				
9. T1 BC	0.15	0.11	0.19*	0.19*	0.68***	0.31***	0.58***	0.21*			
10. T2 BC	0.12	0.17*	0.20*	0.24**	0.16	0.61***	0.15	0.43***	0.08		
11. Age	0.01	0.07	0.11	0.16	0.24**	0.15	0.18*	0.07	0.08	0.19*	
12. Gender	0.11	0.25**	0.08	0.03	0.09	0.11	0.22*	0.15	−0.07	0.09	0.02

*Note: N* = 137.

Abbreviations: AFF = affective empathy, BC = betweenness centrality, CC = closeness centrality, COG = cognitive empathy, DC = degree centrality.

*** *p* < 0.001.

** *p* < 0.01.

* *p* < 0.05.

**FIGURE 1:**
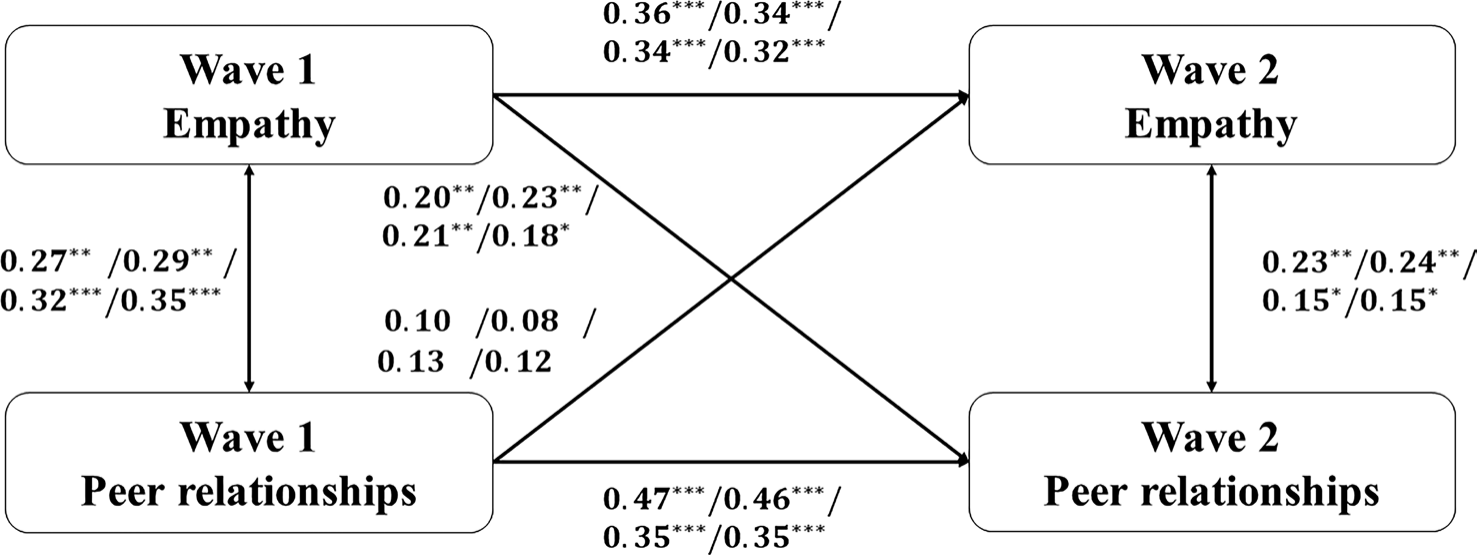
Cross‐lagged panel analysis model of empathy and peer relationship. Results are shown with standardized coefficients in the order of affective empathy to degree centrality, cognitive empathy to degree centrality, affective empathy to closeness centrality and cognitive empathy to closeness centrality. ***p < 0.001, **p < 0.01, *p < 0.05.

Based on the results of our correlation analysis, we conducted a series of cross‐lagged panel analyses as depicted in Figure 1. The results of cross‐lagged panel analyses are partly supported the hypothesis. The cross‐lagged panel analyses concerning affective empathy and degree centrality (*χ* ^2^ = 80.47 with *p* < 0.001 and *df* = 5, RMSEA = 0.00, CFI = 1.00, TLI = 1.00) revealed that there exists a positive and statistically significant relationship between T1 affective empathy and T2 degree centrality (standardized *β* = 0.20, 95%CI = [0.08, 0.33], *p* < 0.01). Conversely, this effect was not observed in the reverse direction (standardized *β* = 0.10, 95%CI = [−0.04, 0.24], *p* > 0.05). Subsequently, we performed a cross‐lagged panel analysis focusing on cognitive empathy and degree centrality (*χ* ^2^ = 79.99 with *p* < 0.001 and *df* = 5, RMSEA = 0.00, CFI = 1.00, TLI = 1.00). The results indicate a significant and positive impact of T1 cognitive empathy on T2 degree centrality (standardized *β* = 0.23, 95%CI = [0.10, 0.33], *p* < 0.01), while the reverse relationship lacks support (standardized *β* = 0.08, 95%CI = [−0.06, 0.21], *p* > 0.05). Moving forward, we conducted a cross‐lagged panel analysis involving affective empathy and closeness centrality (*χ* ^2^ = 57.88 with *p* < 0.001 and *df* = 5, RMSEA = 0.00, CFI = 1.00, TLI = 1.00). The findings illuminate a significant and positive influence of T1 affective empathy on T2 closeness centrality (standardized *β* = 0.21, 95%CI = [0.08, 0.34], *p* < 0.01), whereas the reverse relationship is not substantiated (standardized *β* = 0.13, 95%CI = [−0.01, 0.26], *p* > 0.05). Finally, we conducted a cross‐lagged panel analysis addressing cognitive empathy and closeness centrality (*χ* ^2^ = 53.17 with *p* < 0.001 and *df* = 5, RMSEA = 0.00, CFI = 1.00, TLI = 1.00). This analysis reveals that the effect of T1 cognitive empathy on T2 closeness centrality is positive and significant (standardized *β* = 0.18, 95%CI = [0.04, 0.31], *p* < 0.05), but the reverse relationship is not statistically supported (standardized *β* = 0.12, 95%CI = [−0.02, 0.25], *p* > 0.05). In addition, we did a cross‐lagged panel analysis focusing on cognitive empathy and betweenness centrality, the cross‐lagged effect was not significant.

To investigate the distinct roles of affective empathy and cognitive empathy, we aimed to separate their effects on degree centrality, betweenness centrality and closeness centrality. To account for class‐level effects, we performed hierarchical linear modeling (HLM). Three models were established where three kinds of peer relationships served as dependent variables. The models analyzed the effect size of individual variables (gender, age, and affective empathy, cognitive empathy) on peer relationships (refer to Table 3).

**TABLE 3:** Hierarchical linear model analysis of three kinds of peer relationships.

Model (based on dependent variables)	*X* ^2^ in zero model	Predictor	Coefficients	
			*β*	*p*
T2 degree centrality	24.10***	Age	0.081	0.120
		Gender	0.333	0.360
		T1 affective empathy	0.168	0.136
		T1 cognitive empathy	0.214	0.040
T2 betweenness centrality	10.07*	Age	0.002	0.054
		Gender	0.007	0.406
		T1 affective empathy	−0.001	0.951
		T1 cognitive empathy	0.004	0.126
T2 closeness centrality	22.95***	Age	0.001	0.553
		Gender	0.010	0.151
		T1 affective empathy	0.003	0.170
		T1 cognitive empathy	0.004	0.032

*Note:* To test the hypotheses step by step, we conducted zero model, random‐coefficients regression model, following the methodology of hierarchical linear modeling. *N* = 137; *β* = standardized regression coefficient.

*** *p* < 0.001.

* *p* < 0.05.

First, the HLM analysis revealed that for degree centrality, cognitive empathy could significantly predict degree centrality (*β* = 0.21, *p* < 0.05), while affective empathy did not significantly predict it (*β* = 0.17, *p* = 0.14). Second, the HLM analysis revealed that for betweenness centrality, neither affective empathy (*β* = −0.001, *p* = 0.95) nor cognitive empathy (*β* = 0.004, *p* = 0.13) could predict betweenness centrality. Third, the HLM analysis revealed that for closeness centrality, cognitive empathy could significantly predict closeness centrality (*β* = 0.004, *p* < 0.05), while affective empathy did not significantly predict it (*β* = 0.003, *p* = 0.17).

## 4 Discussion

The present study explores the relationship between empathy in preschoolers and their peer relationships. Employing a two‐wave, 1‐year cross‐lagged panel analysis, our findings reveal a unidirectional influence stemming from preschoolers' empathy toward their peer relationships. Specifically, both affective empathy (T1) and cognitive empathy (T1) are significant predictors of subsequent degree centrality (T2) and closeness centrality (T2), providing support for H1 and H2. Furthermore, HLM results indicate that cognitive empathy (T1), rather than affective empathy (T1), significantly predicts both degree centrality (T2) and closeness centrality (T2).

The unidirectional relationship uncovered by the cross‐lagged panel analysis can be explained by two key factors. First, the origins and developmental trajectories of empathy and peer relationships are distinct. Empathy is rooted in evolutionary selection pressures, particularly in parental care (de Waal 2008) and is considered a stable trait that manifests across different age groups, encompassing both affective and cognitive dimensions (Knafo et al. 2008). Heritability studies have suggested that 27% to 57% of empathy may be inherited (Melchers et al. 2016). In contrast, peer relationships develop as a result of environmental interactions and tend to emerge later than empathy. Second, during the preschool years, the influence of peer relationships on empathy may be limited, possibly due to the early stages of language and communicative development. Kindergarten marks the beginning of peer communication, but children's capacity to engage effectively with peers is still emerging (Chow et al. 2022). Research on adolescents suggests a bidirectional relationship between empathy and peer relationships (Boele et al. 2019; Huang and Su 2014), implying that the effect of peer relationships on empathy becomes more significant as children age. Additionally, the 1‐year, two‐wave design of this study may not fully capture the dynamic developmental changes occurring in preschoolers.

Social network analysis revealed that empathy is significantly associated with two critical centrality measures: degree centrality and closeness centrality. Degree centrality reflects the frequency of peer nominations, with higher values indicating more nominations, while closeness centrality measures how closely connected an individual is to others in the network. Preschoolers with high closeness centrality tend to form connections with peers more quickly. Betweenness centrality, on the other hand, measures how often a preschooler appears on the shortest path between two other preschoolers.

The nature of empathy helps explain these findings. Empathy enables individuals to perceive and understand their peers' emotions (Decety et al. 2015; Yan and Su 2021). Preschoolers with higher levels of empathy are more likely to be attuned to their peers' emotions and thoughts, which facilitates the formation of meaningful peer relationships. This makes empathy a strong predictor of both degree centrality and closeness centrality. However, empathy may have a more limited effect on interactions with peers who are less familiar or emotionally distant (Fowler, Law, and Gaesser 2021). Given the finite nature of empathy as a resource (Hasson et al. 2022), this could explain why empathy does not strongly predict betweenness centrality.

The HLM results suggest that cognitive empathy exerts a stronger influence on peer relationships compared to affective empathy. Empathy is a multidimensional construct. Affective empathy involves automatic and vicarious emotional responses elicited by the emotional experiences of others—it refers to the ability to be directly influenced by others' emotional states by matching or “empathizing” with them (de Waal and Preston 2017). This form of empathy can also be described as affect‐sharing (Gamble et al. 2024). In contrast, cognitive empathy pertains to the capacity to perceive and comprehend the emotional states and perspectives of others (Preston and de Waal 2002; Yan et al. 2020). In terms of social network analysis, degree centrality reflects the number of direct peer nominations an individual receives, while closeness centrality includes both direct and indirect nominations, representing an individual's connections to the entire network. High degree centrality indicates that an individual has many friends, though their precise role within the network may be uncertain—they could either occupy a central position in the broader network or serve as a key figure in a smaller group.

Because affective empathy is automatic and involuntary, it often leads to emotional responses toward a wide range of peers within the social network, fostering connections based on shared emotional experiences (Eisenberg and Fabes 1995). However, while emotional resonance forms the foundation of peer relationships, perspective‐taking, which is part of cognitive empathy, plays a more significant role in developing and sustaining these relationships (Georgiou, Kimonis, and Fanti 2019), particularly in preschoolers. Cognitive empathy, unlike affective empathy, requires conscious effort, control, and active engagement (Bensalah, Caillies, and Anduze 2015), which are critical for perspective‐taking. Wellman, Cross, and Watson (2001) found that around age five, children experience a key period in the development of Theory of Mind, during which they become better able to understand their own and others' psychological states. Gonzalez‐Liencres, Shamay‐Tsoory, and Brüne (2013) further elaborated that cognitive empathy is rooted in Theory of Mind processes, allowing individuals to infer motives, predict behaviors, and gain deeper self and social understanding. This enhanced understanding promotes greater social sensitivity and adaptability, fostering more extensive peer relationships. Moreover, empathy is not distributed equally across all peers—we tend to empathize more with those to whom we are closer (Hasson et al. 2022). Therefore, cognitive empathy, rather than affective empathy, shows a stronger association with both degree centrality and closeness centrality in peer relationships.

## 5 Limitations and Implications for Further Research

Several limitations of this study warrant consideration. First, while the current research focuses exclusively on a longitudinal analysis of preschoolers, it does not explore the relationship between empathy and peer relationships in adolescents and adults. Future research is necessary to investigate whether these causal connections vary across different age groups. Second, this study relies solely on self‐reported data from preschoolers, which introduces potential bias due to the limited perspective provided by a single data source. To improve the robustness of future findings, it would be beneficial to incorporate additional data sources, such as peer assessments and classroom observations, allowing for a more comprehensive analysis. Third, the peer nominations used in this study may not fully capture the complexities of preschoolers' peer relationships. Future studies should aim to differentiate between various types of peer relationships to provide a more nuanced understanding. Finally, several potential confounding variables, such as parental education levels and income, were not considered in this study. Future research should account for these and other contextual factors to better understand their influence on the relationship between empathy and peer relationships.

## Conflicts of Interest

The authors declare no conflicts of interest.
